# A modified surgical technique for children recurrent popliteal cyst: the repair method of using medial head of gastrocnemius tendon flap

**DOI:** 10.3389/fped.2025.1597186

**Published:** 2025-09-19

**Authors:** Jun Li, Zhixiang Xiao, Jingyi Chen, Shaohua He, Di Xu, Jianglong Chen, Yingquan Kang

**Affiliations:** ^1^ Department of Pediatric Orthopedics, Fuzhou University Affiliated Provincial Hospital, Shengli Clinical Medical College, Fujian Medical University, Fuzhou, China; ^2^ Department of Pediatric Surgery, Fuzhou University Affiliated Provincial Hospital, Shengli Clinical Medical College, Fujian Medical University, Fuzhou, China

**Keywords:** gastrocnemius medial head tendon flap, repair, recurrent, popliteal cyst, children

## Abstract

**Objective:**

To investigate the efficacy of gastrocnemius medial head tendon flap repair in the treatment of recurrent popliteal cysts in children.

**Methods:**

A retrospective analysis was conducted on the data of 65 cases of recurrent popliteal cysts in children admitted to our department from January 2014 to December 2023. Preoperatively, the popliteal cysts were graded according to the Rauschning and Lindgren grading: Grade 0 in 1 case, Grade I in 10 cases, Grade II in 23 cases, and Grade III in 31 cases. The preoperative Lysholm score of the affected knee joint was (71.79 ± 5.95) points. During the operation, after the removal of the popliteal cyst, the medial head of gastrocnemius tendon flap repair was performed to close the joint capsule hernia orifice. The clinical efficacy was assessed by comparing the Rauschning and Lindgren popliteal cyst grading and the Lysholm score of the affected knee joint preoperatively and at 1, 6, 12, and 24 months postoperatively.

**Results:**

All 65 cases were followed up. No significant neurological complications occurred postoperatively in any case. At the last follow-up, no limitations in knee joint motion were observed. The surgical duration ranged from 30 to 165 min, with a mean of (74.18 ± 28.16) minutes; intraoperative blood loss ranged from 10 to 30 ml, with a mean of (17.43 ± 5.36) ml; and the postoperative hospital stay ranged from 2 to 6 days, with a mean of (3.03 ± 1.91) days. At 1, 6, 12, and 24 months postoperatively, the Rauschning and Lindgren popliteal cyst grading and the Lysholm score of the affected knee joint showed significant improvement compared with preoperative values, with statistically significant differences (*P* < 0.05). The grading and scores at 6, 12, and 24 months postoperatively were significantly better than those at 1 month postoperatively, with statistically significant differences (*P* < 0.05). However, no significant differences were observed in the grading and scores between 6, 12, and 24 months postoperatively (*P* > 0.05). No recurrence was detected in any case during the regular follow-up examinations.

**Conclusion:**

The gastrocnemius medial head tendon flap repair can effectively reinforce the hernial orifice between the popliteal cyst and the knee joint cavity, achieving good therapeutic efficacy in treating recurrent popliteal cysts in children.

## Introduction

Popliteal cysts were first described by Baker in 1877 and are a common condition of the knee joint in children (1). The cause and pathophysiology of popliteal cysts in children is not yet fully understood. The most frequently mentioned theory in previous studies has been that BC is caused by the connection between the cyst and the joint space (2). Traditional surgical procedures often involve open excision of the cyst followed by direct ligation and suturing of the neck of the cyst hernia sac, but a high recurrence rate after surgery has been frequently reported in the literature (3). In recent years, our department has modified local repair techniques by using a gastrocnemius medial head tendon flap repair method to cover the hernial orifice of the posterior knee joint capsule. We performed surgery on 65 children with recurrent popliteal cysts after excision, and the outcomes were satisfactory. A report is presented as follows.

## Materials and methods

### Patients and design

This study retrospectively analyzed the clinical data of 65 children who underwent surgical treatment in our department from January 2014 to December 2023. Among them, there were 55 boys and 10 girls. Age ranged from 3 to 11 years, with an average of 6.11 ± 2.06 years. 32 cases were on the left side, and 33 cases were on the right side. Postoperative recurrence duration were 3.00 (3.00, 4.00) months and all cases denied a history of trauma. Preoperative knee joint MRI examinations were performed, and the popliteal cysts were classified according to the degree of knee joint pain, swelling, and limited range of motion based on the Rauschning and Lindgren grading: Grade 0 in 1case, Grade I in 10 cases, Grade II in 23 cases, Grade III in 31 cases. The preoperative Lysholm score for the affected knee joint was 71.79 ± 5.95 points (Table 1). During surgery, after excising the recurrent cyst, the gastrocnemius medial head tendon flap repair was performed to repair the hernial orifice of the posterior knee joint capsule.

**Table 1:** Baseline patient characteristics.

Variables	Statistic
Age (year)	6.11 ± 2.06
Postoperative recurrence duration (month)	3.00 (3.00, 4.00)
Gender (*n*,%)	
Male	55 (84.62)
Female	10 (15.38)
Affected side (*n*,%)	
Left	32 (49.23)
Right	33 (50.77)

Inclusion criteria: 1. The patient was diagnosed with popliteal cyst at the first visit and it recurred after one or more surgeries (the definition of the recurrent popliteal cysts); 2. Under the age of 18.

Exclusion criteria: 1. Initial surgery; 2. Over the age of 18.

### Surgical techniques

**Figure 1:**
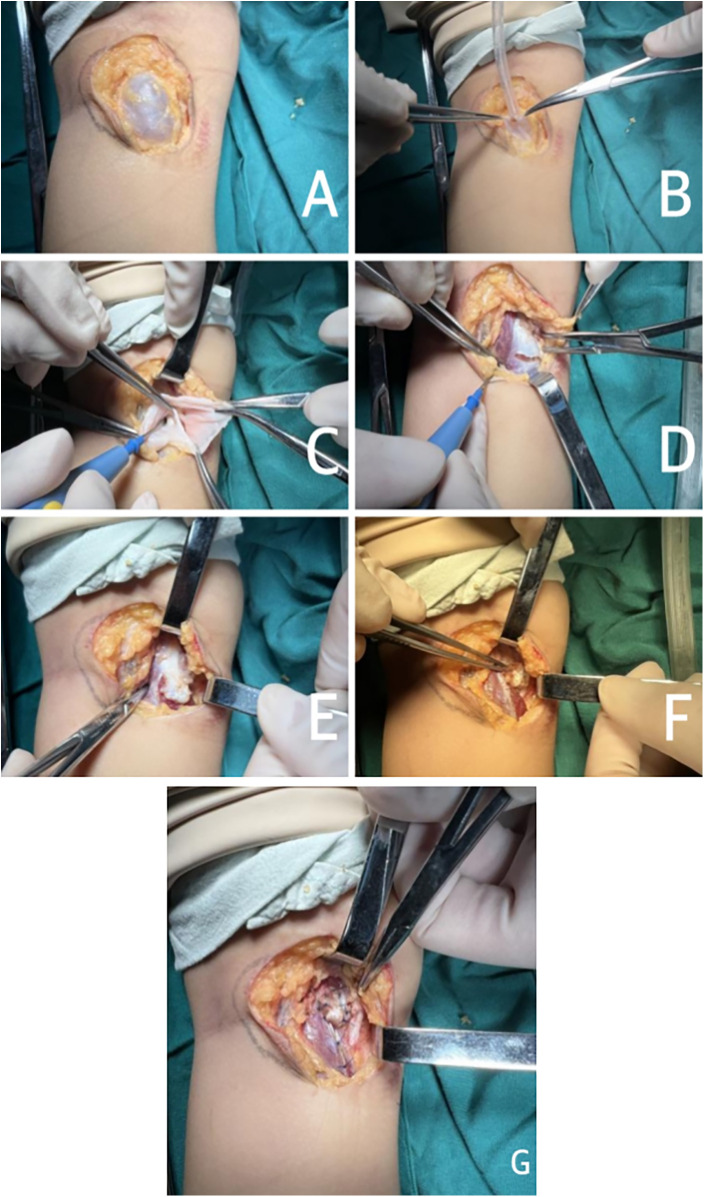
Gastrocnemius medial head tendon flap repair for treating recurrent popliteal cysts. **(A)** Careful exposure of cyst; **(B)** Opening the cyst in advance and draining the fluid; **(C)** The cyst communicating with the posterior knee joint cavity; **(D)** Spliting the medial head of gastrocnemius tendon with electrotome; **(E)** Forming a thin tendon flap of the medial head of the gastrocnemius tendon; **(F)** Fliping up the medial head tendon flap of the gastrocnemius muscle; **(G)** The hernia orifice was repaired using the medial head tendon flap of gastrocnemius tendon.

After general anesthesia, the child was placed in the prone position, and the area was sterlized. An “S” shaped incision of the medial popliteal fossa, 3–4 cm in length, was made. The skin, subcutaneous tissue, and deep fascia were incised layer by layer, carefully exposing the recurrent cyst (Figure 1A), while taking care to protect the surrounding blood vessels and nerves. The base of the cyst was seen extending into the posterior knee joint cavity. The cyst was opened in advance, and the cyst fluid was aspirated using a suction device (Figure 1B). The cyst wall was separated to the base, and the excess cyst wall was excised to reveal the hernial orifice communicating with the joint capsule (Figure 1C). A thin layer of tendon from the medial head of the adjacent gastrocnemius was split (Figure 1D) to form a thin tendon flap slightly larger than the base (Figure 1E), which was then folded over and circularly sutured to repair the hernial orifice (Figures 1F,G). The wound was irrigated, and hemostasis was thoroughly achieved. The incision was closed in layers with absorbable sutures. The excised cyst was sent for pathological examination. Postoperatively, the limb was immobilized with a plaster cast for 3 weeks.

### Data collection

Follow-up was conducted through outpatient reviews and telephone inquiries. Within the first year postoperative, the knee joint was examined every six months using ultrasound, and annually thereafter with ultrasound. Based on the preoperative and postoperative Rauschning and Lindgren popliteal cyst grading and the Lysholm score assessed by the surgeon of the affected knee joint, the improvement of the children's clinical symptoms was assessed.

### Statistical analysis

Data analysis was conducted using SPSS 23.0 statistical software. The Shapiro–Wilk test was used to assess the normality of the measurement data. If the *P*-value of the Shapiro–Wilk test was greater than 0.05, the measurement data were considered to follow a normal distribution. Otherwise, the measurement data were considered not to follow a normal distribution. Measurement data that met the normal distribution were expressed as *x* *±* *s*, while those that did not meet the normal distribution were expressed as M (Q1, Q3). Count data were compared as percentages (%). The Friedman test was used for the comparison of grades before and after surgery. For significant variables, pairwise comparisons were conducted using the Bonferroni method. Repeated measures analysis was used for the comparison of scores before and after surgery, and pairwise comparisons for significant variables were conducted using the Turkey method. A *P*-value less than 0.05 was considered statistically significant.

## Results

### The baseline patient characteristics

#### The perioperative period general data

In 65 cases, postoperative pathology confirmed all were recurrences of popliteal cysts. All postoperative incisions healed well, with no significant neurological complications such as sensory or motor dysfunctions observed. All cases had follow-ups. At the last follow-up, no limitations in knee joint movement were found. The operation time was 30–165 min, with an average of 74.18 ± 28.16 min. Intraoperative blood loss was 10–30 ml, with an average of 17.43 ± 5.36 ml. Postoperative hospitalization days were 2–6 d, with an average of 3.03 ± 1.91 d.

#### The rauschning and lindgren popliteal cyst grading and the lysholm score results

At 1, 6, 12, and 24 months postoperatively, the Rauschning and Lindgren popliteal cyst grading and the Lysholm score of the affected knee showed significant improvement compared to the preoperative levels, with differences being statistically significant (*P* < 0.05). At 6, 12, and 24 months postoperatively, the grading and score also demonstrated improvement compared to those at 1 month postoperatively, with differences being statistically significant (*P* < 0.05). However, the results of grading and score at 1 month, 6 months and 24 months after surgery showed no significant difference among all groups (*P* > 0.05) (Tables 2, 3).

**Table 2:** Preoperative and postoperative Rauschning and Lindgren popliteal cyst grading.

Variables	Grade 0	Grade I	Grade II	Grade III
Preoperative	1 (1.54)	10 (15.38)	23 (35.38)	31 (47.69)
1 month postoperative^a^	9 (13.85)	29 (44.62)	20 (30.77)	7 (10.77)
6 months postoperative^a^^,^^b^	18 (27.69)	43 (66.15)	4 (6.15)	0 (0.00)
12 months postoperative^a^^,^^b^	18 (27.69)	43 (66.15)	4 (6.15)	0 (0.00)
24 months postoperative^a^^,^^b^	19 (29.23)	40 (61.54)	5 (7.69)	1 (1.54)
*Z*	122.911			
*P*	<0.001*			

^a^ Compared with preoperative levels, the differences were statistically significant (*P* < 0.05).

^b^ Compared with the levels at 1 month postoperatively, the differences were statistically significant (*P* < 0.05).

*The differences were statistically significant (*P* < 0.05).

**Table 3:** Preoperative and postoperative Lysholm score of the affected knee.

Variables	Preoperative	1 month postoperative^a^	6 months postoperative^a^^,^^b^	12 months postoperative^a^^,^^b^	24 months postoperative^a^^,^^b^
Lysholm score	71.97 ± 5.95	78.35 ± 5.26	84.91 ± 4.92	85.15 ± 4.58	85.57 ± 4.97
*F*	86.687				
*P*	<0.001*				

^a^ Compared with preoperative levels, the differences were statistically significant (*P* < 0.05).

^b^ Compared with the levels at 1 month postoperatively, the differences were statistically significant (*P* < 0.05).

*The differences were statistically significant (*P* < 0.05).

#### Typical case

**Figure 2 AND 3:**
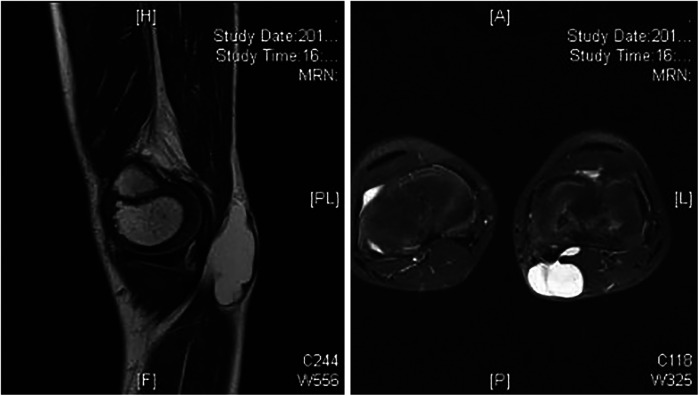
Preoperative MR sagittal and transverse views show multicystic lesions around the medial head of the gastrocnemius.

**Figure 4 AND 5:**
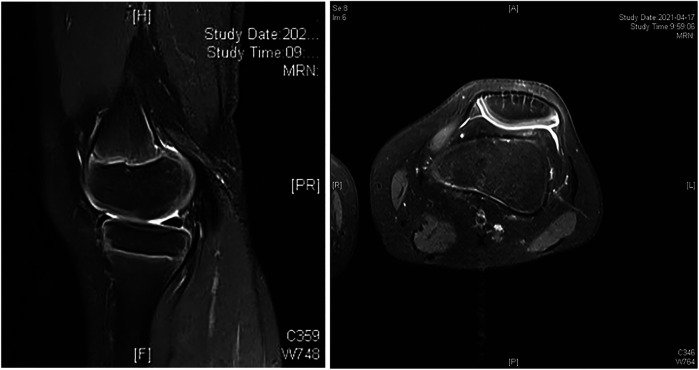
MR sagittal and transverse views show no recurrence at 3 years postoperative.

A 9-year-old boy was admitted with the chief complaint of recurrent popliteal cyst. Preoperative knee joint MRI indicated: cyst around the medial head of the left knee joint (Figures 2, 3). He underwent excision of left popliteal cyst combined with gastrocnemius medial head tendon flap repair. Postoperative 3 years follow-up knee joint MRI showed no recurrence (Figures 4, 5).

## Discussion

The etiology of popliteal cysts is currently thought to be possibly related to a unidirectional valve mechanism within the knee joint. Essentially, it is an enlarged gastrocnemius-semimembranosus bursa (GSB), and the presence of a unidirectional valve causes fluid to flow from the knee joint cavity to the bursa, which is a fundamental factor in the formation and persistence of popliteal cysts (4). Due to the action of the gastrocnemius and semitendinosus, during flexion the “valve” opens and the synovial fluid under pressure moves into the bursa; during extension the “valve” closes due to the tension of these muscles and the fluid remains trapped inside the bursa (5). The related clinical symptoms and the size and location of the cyst are closely related, and common symptoms include: popliteal mass or swelling, knee joint effusion, pain, thrombophlebitis, knee joint locking and so on (6).

Popliteal cysts in children differ from those in adults (7). They typically have no history of trauma, mostly primary, and usually have no lesions within the joint cavity (7–9). In children, popliteal cysts tend to occur most frequently in those aged 4–7 years (10). De Maeseneer conducted an epidemiological survey on popliteal cysts in children, finding an incidence rate of 6.3% (11). Seil found that 2.4% of children under 15 years old with popliteal cysts are asymptomatic (12). Whether the popliteal cysts are connected with the articular cavity is controversial. It was previously believed that popliteal cysts in children did not communicate with the knee joint cavity (13). However, with the widespread use of ultrasound, MRI, and arthrography, numerous studies have believed that most popliteal cysts in children are connected to the knee joint cavity (14). Due to the communication between the cyst and the joint cavity, incomplete or difficult repair of the rupture between them during the initial surgery is a significant reason for postoperative cyst recurrence.

The classic surgical procedure for children's popliteal cysts is open cyst excision, closing the channel between the cyst and the joint cavity. However, because the cyst wall is difficult to separate and the base of the cyst is not easily ligated thoroughly. Literature reports a recurrence rate of up to 50%–63% for this procedure. In 2014, Ningbo and others proposed the internal purse-string suture method for treating 26 cases of pediatric communicating popliteal cysts (15). This method involves preemptively opening the cyst and using a technique similar to high ligation of a pediatric hernia sac at the cyst's root for internal purse-string suturing, yielding satisfactory results. Research by Su and others indicates that internal drainage of the cyst combined with cyst wall resection under arthroscopy of the knee joint can reduce the recurrence rate of cysts compared to simple internal drainage of the cyst, although it simultaneously increases the incidence of perioperative complications (16). Huang used a technique to manage the recalcitrant popliteal cysts through posterior capsule reconstruction with a double-layered tensor fascia lata graft. This technique apparently offers very secure anchorage plus double coverage (17).

Current strategies for recurrent cysts emphasize addressing the fundamental communication pathway. Traditional open excision alone has been associated with higher recurrence rates, leading to the development of various techniques aimed at securely closing the neck of the cyst. These include open techniques with internal purse-string suturing or weaving sutures to reinforce the potential space between the semimembranosus and semitendinosus tendons, and arthroscopic procedures such as cystectomy, internal drainage, and widening of the valvular opening to eliminate the one-way valve mechanism. Recent studies suggest that arthroscopic management, while potentially more technically demanding, can offer advantages in terms of lower recurrence rates and better visualization of intra-articular structures, though robust comparative studies specifically in recurrent pediatric cases are limited.

In this study, we used a modified local repair technique to treat recurrent popliteal cysts in children. Intraoperatively, it was found that the cyst cavity was filled with clear yellow fluid, and the base of the cyst was connected to the knee joint cavity, which also confirmed the views of modern literature. How to better manage the channel between the cyst and the joint cavity during surgery is key to preventing recurrence. We adopted the technique of splitting and suturing the gastrocnemius medial head tendon flap to reinforce the knee joint hernial orifice. We believe that this suturing method can minimize the residual volume of the posteriorly protruding joint capsule. Additionally, the gastrocnemius medial head tendon flap fixed at the hernial orifice can further strengthen the weakened posterior joint capsule after cyst excision, resisting the intra-articular pressure, and etiologically blocking the unidirectional outflow of fluid from the knee joint cavity, avoiding excessive tension caused by direct suturing of the hernial orifice, and postoperative movement caused tearing at the suture site, leading to the reopening of the hernial orifice and recurrence of the cyst (18). Compared with tensor fascia lata graft reconstruction of the posterior capsule, the gastrocnemius medial head tendon flap repair is less invasive and does not require additional lateral thigh incision. In this series of cases, the recurrence cases showed significant adhesion at the surgical site, making cyst dissection difficult and the base unidentifiable. Direct suture repair of the joint capsule hernial orifice was challenging. The use of the gastrocnemius medial head tendon flap for repair effectively avoided these issues, simplified the surgical procedure, shortened the surgery time, and reduced blood loss. Through follow-up, none of the 65 children showed cyst recurrence. And the postoperative knee joint Lysholm score and the Rauschning and Lindgren popliteal cyst grading improved compared to preoperative, with the difference being statistically significant at 1 month, 6 months, 12 months, 24 months (*P* < 0.05). However, there was no difference in score and grading between all the groups after surgery (*P* > 0.05). These results indicate that the treatment of recurrent popliteal cyst with gastrocnemius medial head tendon flap repair is effective in the short-term postoperative follow-up.

The purpose of plaster fixation after popliteal cyst surgery is to keep the children's knee joint at rest, preventing increased intra-articular pressure due to early postoperative movement, which is beneficial for the solid healing of the gastrocnemius medial head tendon flap repair at the hernial orifice, effectively preventing early cyst recurrence. During long-term postoperative follow-up, we found that partial splitting of the gastrocnemius medial head tendon flap and postoperative plaster fixation did not affect the knee joint function of the children.

Of course, there are also shortcomings in this study. This study is a single-center retrospective case analysis with a small sample size. It awaits further research and validation through multi-center, large-sample, long-term follow, prospective studies. The gastrocnemius medial head tendon flap repair technique for treating recurrent popliteal cysts in children has not yet been widely adopted, and the long-term postoperative efficacy requires further follow-up for validation. In future studies, the sample size will be increased, and the follow-up period will be extended to further validate the efficacy of this surgical procedure.

## Conclusion

In summary, the gastrocnemius medial head tendon flap repair can effectively reinforce the repair between the popliteal cyst and the hernial orifice of the knee joint cavity, achieving good results in treating recurrent popliteal cysts in children.

## Data Availability

The raw data supporting the conclusions of this article will be made available by the authors, without undue reservation.

## References

[1] Baker WM. On the formation of synovial cysts in the leg in connection with disease of the knee-joint. Clin Orthop Relat Res. (1994) 1994 Feb(299):2–10. 8119018

[2] Han DY Ryu KN Park JS Jin W Park SY Yun SJ. The prevalence of Baker cyst in relation to the arrangement pattern between the medial head of gastrocnemius tendon and the semimembranosus tendon. Eur Radiol. (2020) 30(3):1544–53. 10.1007/s00330-019-06472-6 31811432

[3] Saylik M Gökkuş K. Treatment of Baker cyst, by using open posterior cystectomy and supine arthroscopy on recalcitrant cases (103 knees). BMC Musculoskelet Disord. (2016) 17(1):435. 10.1186/s12891-016-1291-5 27756267 PMC5069796

[4] Park GY Kwon DR Kwon DG. Clinical, radiographic, and ultrasound findings between simple and complicated Baker’s cysts. Am J Phys Med Rehabil. (2020) 99(1):7–12. 10.1097/PHM.0000000000001263 31335340

[5] Abate M Di Carlo L Di Iorio A Salini V. Baker’s cyst with knee osteoarthritis: clinical and therapeutic implications. Med Princ Pract. (2021) 30(6):585–91. 10.1159/000518792 34348320 PMC8739941

[6] Irismetov ME Tursunov KK Khudayberdiev KT Tursunova MA Roitblat Y. Diagnostics and surgical treatment of ruptured Baker’s cysts: a prospective study. J Clin Orthop Trauma. (2024) 58:102792. 10.1016/j.jcot.2024.102792 39564593 PMC11570460

[7] Poboży T Konarski W Piotrowska-Lis K Domanska J Pobozy K Kielar M. Basic differences and most common findings in ultrasound examinations of musculoskeletal system in children: a narrative literature review. Healthcare. (2022) 10(10):2010. 10.3390/healthcare10102010 36292459 PMC9602487

[8] Tessier S Murphy RJ Peake KN Longo S Erickson-Parsons LA. Baker’s cyst: a harbinger of systemic joint disease in children. Clin Pediatr (Phila). (2024) 63(3):419–22. 10.1177/00099228231175467 37208898

[9] Zhu J Xiang D Yang S Xiang L Liu X. Arthroscopic internal drainage of popliteal cysts with cyst wall resection in pediatric patients. Pak J Med Sci. (2022) 38(8):2278–83. 10.12669/pjms.38.8.5354 36415283 PMC9676583

[10] Leib AD Roshan A Foris LA Varacallo MA. Baker’s Cyst[M]//StatPearls. Treasure Island, FL: StatPearls Publishing (2025). 28613525

[11] De Maeseneer M Debaere C Desprechins B Osteaux M. Popliteal cysts in children: prevalence, appearance and associated findings at MR imaging. Pediatr Radiol. (1999) 29(8):605–9. 10.1007/s002470050659 10415188

[12] Seil R Rupp S Jochum P Schofer O Mischo B Kohn D. Prevalence of popliteal cysts in children. A sonographic study and review of the literature. Arch Orthop Trauma Surg. (1999) 119(1-2):73–5. 10.1007/s004020050358 10076949

[13] You C Cheng Z Xia Y Deng C Zhou Y. Comparison of arthroscopic internal drainage and open excision for the treatment of popliteal cysts. BMC Musculoskelet Disord. (2022) 23(1):732. 10.1186/s12891-022-05658-2 35907946 PMC9338577

[14] Zhou Y Yang Q Kang J He S. Analysis of the efficacy of triple-channel minimally invasive knee arthroscopy in treating popliteal cysts in children. BMC Surg. (2024) 24(1):326. 10.1186/s12893-024-02620-y 39443918 PMC11515641

[15] Ning B Yao J Yuan Y Zhang Y Bai C Chen W Treatment of popliteal cyst in children by internal purse-string suture. Chin J Pediatr Surg. (2014) 35(1):24–7. 10.3760/cma.j.issn.0253-3006.2014.01.006

[16] Su C da Kuang S Zhao X Li YS Xiong YL Gao SG. Clinical outcome of arthroscopic internal drainage of popliteal cysts with or without cyst wall resection. BMC Musculoskelet Disord. (2020) 21(1):440. 10.1186/s12891-020-03453-5 32631287 PMC7339393

[17] Huang CK Hong CK Kuan FC Su WR Hsu KL. Double-layered reconstruction of the posterior capsule in a recalcitrant Baker’s cyst: a case report. J Orthop Sci. (2024) 29(1):390–3. 10.1016/j.jos.2022.04.005 35487800

[18] Yang L Tang X Li Q Chen G Fu W Jiang H Arthroscopic treatment combined with repair of joint capsule using tendon flap of medial head of gastrocnemius muscle after resection of popliteal cyst. Chin J Reparative Reconstr Surg. (2015) 29(12):1462–5. 10.7507/1002-1892.20150313 27044210

